# Physical training for patients with depression and anxiety - a randomized controlled study

**DOI:** 10.1192/j.eurpsy.2021.1828

**Published:** 2021-08-13

**Authors:** Q. Zhai, Y. Freund-Levi, A. Horn, A.-C. Fridenberger, E. Lager, S. Montgomery, J. Persson

**Affiliations:** 1 School Of Medical Sciences, Örebro University, Örebro, Sweden; 2 Psychiatry, Örebro University Hospital, Örebro, Sweden; 3 Department Of Neurology, Care Sciences And Society, Karolinska Institutet, Stockholm, Sweden; 4 School Of Law, Psychology And Social Work, Örebro University, Örebro, Sweden

**Keywords:** Depression, Anxiety, Exercise, cognitive functions

## Abstract

**Introduction:**

Pharmaceutical treatment and psychotherapy constitute the most common treatment methods for depression and anxiety. Physical training has been shown to have comparable effect to cognitive behavioral therapy in treatment of mild to moderate depression and anxiety. Physically active individuals also show lower risks to develop depression and relapse in depression.

**Objectives:**

The objectives are to evaluate how physical activity can affect depressive and anxiety symptoms, by examining biomarkers in the blood and from the gut and also by measuring cognitive functions. Hopefully, this can lead to new treatment strategies for patients with depression and anxiety.

**Methods:**

102 patients are randomized to two groups and undergo 12 weeks intervention as add-on to standard outpatient psychiatric treatment. The first group will participate in physical training three times per week and the other group will receive relaxation therapy on a weekly basis. Daily activity intensity will be measured before and at the last week of intervention with an accelerometer. Blood and faeces sample collection, symptom grading by clinician together with self-rating scales and cognitive screening will be performed at baseline, week 12 and one year of follow-up. The cognitive screenings are performed digitally in cooperation with Mindmore.

**Results:**

The RCT is currently recruiting patients at the Department of Psychiatry of Örebro University Hospital.

**Conclusions:**

The project aims to be holistic in its approach, combining the defining clinical psychiatric symptoms in patients who have both depression and anxiety with the finding and evaluation of new biomarkers from blood and gut to improve cognitive functions.

**Disclosure:**

No significant relationships.

